# Diagnosing Pulmonary Embolism Using Point-of-Care Ultrasound in a Patient With Malingering and Coccidioidomycosis Infection

**DOI:** 10.7759/cureus.34288

**Published:** 2023-01-27

**Authors:** Uday Chauhan, Pal Athwal, Mohammed Elhassan

**Affiliations:** 1 Internal Medicine, Saint Agnes Medical Center, Fresno, USA

**Keywords:** malingering, computed tomography pulmonary angiography, pulmonary coccidioidomycosis, point-of-care-ultrasound, pulmonary embolism (pe)

## Abstract

We report a case of a 41-year-old male diagnosed with pulmonary coccidioidomycosis and pulmonary embolism (PE) based on a point-of-care ultrasound (POCUS) finding who was suspected to be malingering for right-sided chest pain considering his psychiatric history. POCUS was performed and showed right ventricular strain with a D-shaped left ventricle and B-lines with subpleural consolidations, and PE was confirmed with computed tomography pulmonary angiography. No other risk factors for PE were found except for coccidioidomycosis. The patient was treated with apixaban and fluconazole and discharged in stable condition. We discuss the usefulness of POCUS in diagnosing PE and the very rare association between coccidioidomycosis and PE.

## Introduction

Pulmonary embolism (PE) is defined as a form of venous thromboembolism (VTE) obstructing pulmonary vasculature [1]. It continues to be a leading cause of hospital morbidity and mortality. There are approximately 60,000-100,000 deaths from PE in the United States alone [2]. The signs and symptoms are nonspecific, and presentations of PE can vary from asymptomatic to massive PE with hemodynamic instability associated with poor outcomes [3]. In patients with psychiatric histories who complain of chest pain, malingering can potentially delay the diagnosis of organic causes and possibly lead to adverse events. Serious diagnoses, including PE, should always be considered in these patients [4]. In such cases, point-of-care ultrasound (POCUS) performed at the bedside can be useful diagnostically [5]. It might reveal signs of right ventricular (RV) strain, which can raise clinical suspicion for the diagnosis of PE and prompt appropriate diagnostic tests, including computed tomography pulmonary angiography (CTPA) [6]. Coccidioidomycosis (also known as Valley Fever) is prevalent in Central and South America. Coccidioides immitis and Coccidioides posadasii are the two species causing infections in humans [7]. The association between PE and coccidioidomycosis in patients without risk factors for thromboembolism is not well established in the literature. A literature search at the time of writing the paper and with the assistance of a librarian revealed only one case series of five patients that reported this specific association [8].

Our case report demonstrates how POCUS, a quick and safe diagnostic test, can assist in sorting out the diagnostic possibilities of chest pain in patients with potential diagnoses of malingering. It also demonstrates the potential association between coccidioidomycosis and VTE in patients without detectable risk factors for acquired or inherited thromboembolism.

## Case presentation

A 41-year-old African American male with a past medical history of schizophrenia, polysubstance use with alcohol and methamphetamine, and homelessness presented to the hospital with auditory and visual hallucinations and homicidal ideation. He also reported intermittent fever, cough, right-sided chest pain, and dyspnea. Family history was unremarkable, with no history of contact with sick people recently. There was no recent travel history either.

Initial vital signs on admission included a temperature of 38.2 °C, blood pressure of 120/74 mmHg, heart rate of 105 beats per minute, respiratory rate of 18 breaths per minute, and oxygen saturation of 98% on ambient air. Physical examination revealed a thin male without apparent distress but appeared disheveled and mildly anxious, fully oriented with normal speech, and without adenopathy. Respiratory examination showed right lung crackles, but the rest of the systemic examination was normal.

**Figure 1:**
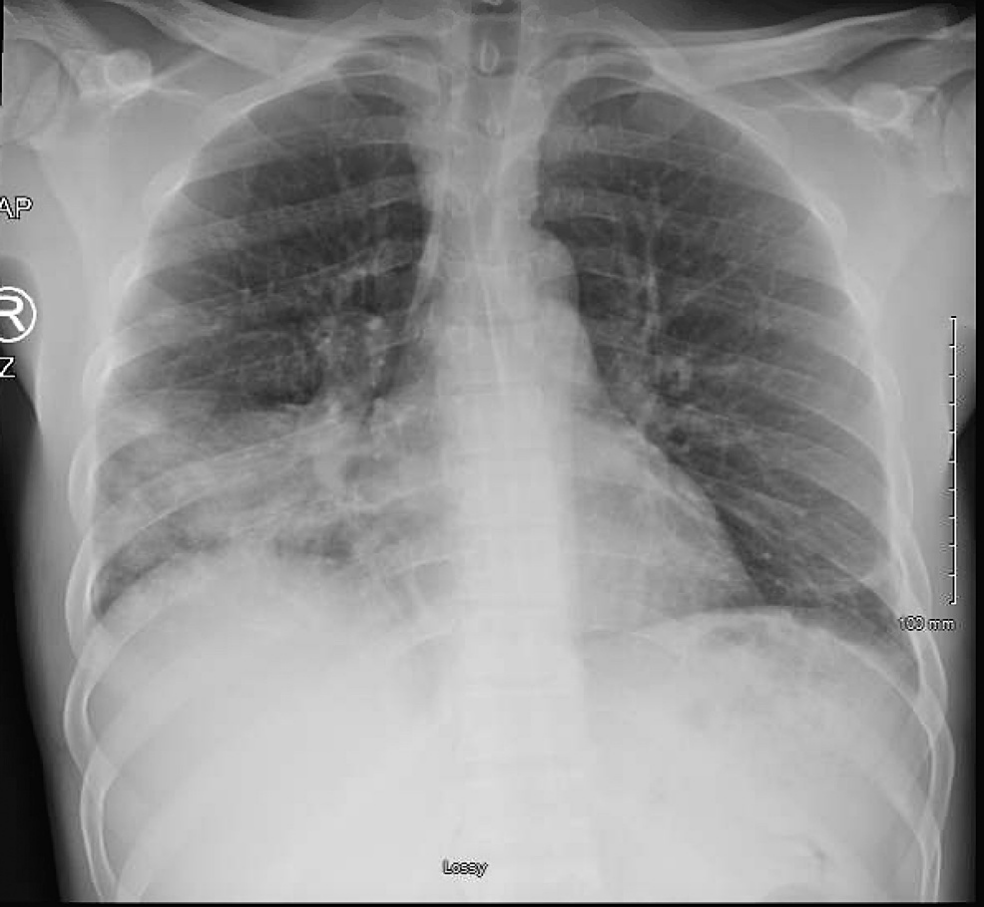
A chest X-ray showing right-sided lower lobe consolidation with an air bronchogram.

Laboratory data showed normal metabolic panel and serum lactate, no anemia or leukocytosis but mild eosinophilia at 0.52 thousand/mcL. The urine drug screen was positive for methamphetamine. Both polymerase chain reactions for SARS-CoV-2 and HIV were negative. The tuberculosis QuantiFERON test was also negative. An anterior-posterior chest X-ray revealed a right lower lobe consolidation with an air bronchogram (Figure 1).

**Figure 2:**
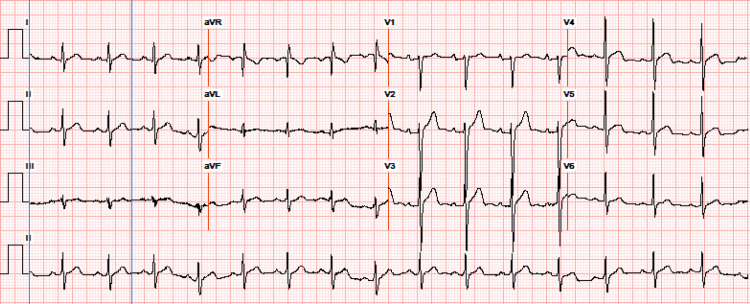
Electrocardiogram showing normal sinus rhythm without acute ischemic changes.

A cardiac workup ruled out acute coronary syndrome and ECG showed regular rate and rhythm without any change from baseline, as shown in Figure 2. 

**Figure 3:**
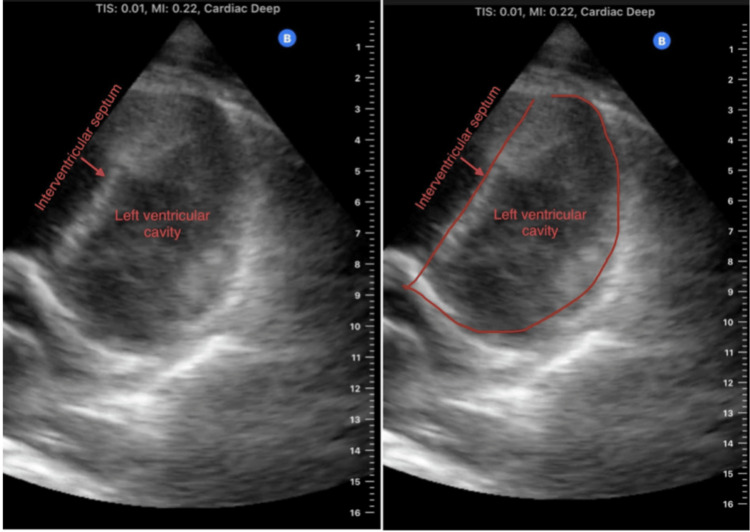
Cardiac POCUS using the short-axis left parasternal view showing flattening of the interventricular septum and giving rise to the D-shaped left ventricle (D-sign), which is seen in patients with right ventricular strain. POCUS, point-of-care ultrasound

**Figure 4:**
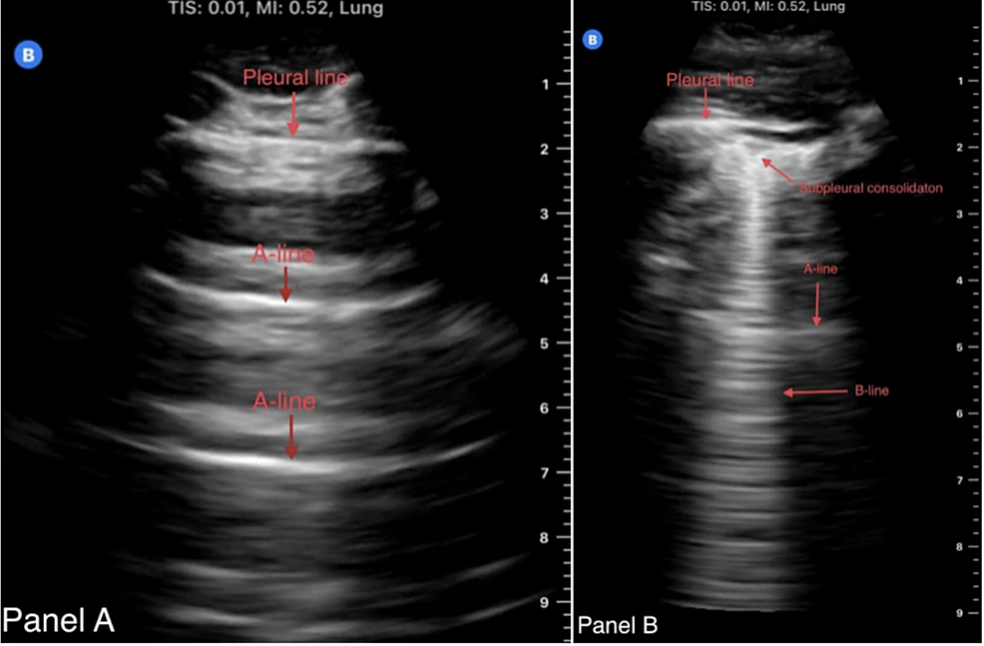
Panel A shows a patient's normal lung POCUS in the left upper lung anteriorly before the development of the new left-sided pleuritic chest pain. Panel B shows lung POCUS during the evaluation of the new left-sided pleuritic chest pain around the same lung area, and it shows new triangular-shaped subpleural consolidation with an associated B-line suggesting new interstitial process, including pulmonary embolism. POCUS, point-of-care ultrasound

The patient was initially treated for community-acquired pneumonia with antibiotics without clinical response. Coccidioidomycosis IgG and IgM antibodies came back positive, and the patient was started on fluconazole. Psychiatric consultation was also obtained, given the patient’s symptoms, and showed that the patient was no more suicidal but was homeless and said, “I know what to say to stay in the hospital.” The psychiatrist suspected malingering as a potential cause of his psychiatric symptoms. Two days later, the patient started to complain of new onset of left-sided chest pain, away from the site of his original right-sided pleuritic chest pain from pneumonia. Repeat ECG and serum troponin were unremarkable. The chest pain was initially attributed to either mild left-sided involvement of the patient's coccidioidomycosis pneumonia or malingering, but POCUS was performed at the bedside for further evaluation. Unexpectedly, it showed RV strain with D-shaped left ventricle and lung B-lines with subpleural consolidations, not previously seen on admission (Figures 3-4).

**Figure 5:**
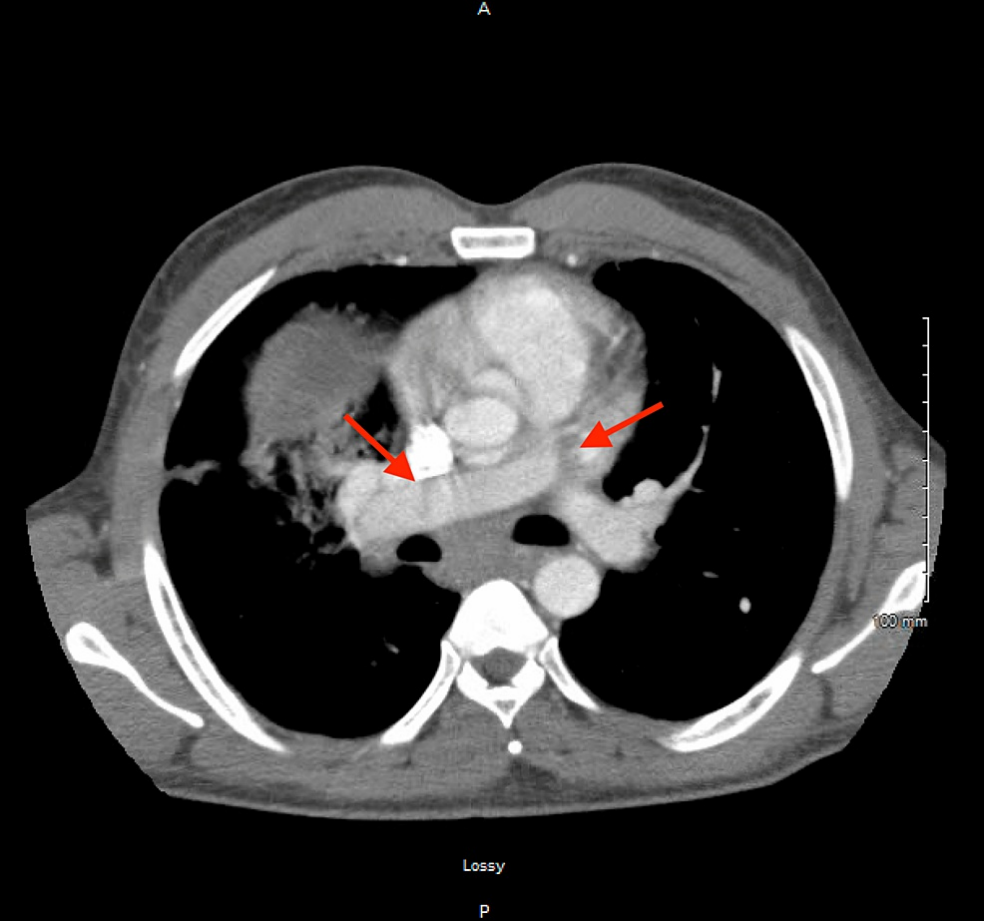
CTPA showing bilateral pulmonary emboli (red arrows). CTPA, computed tomography pulmonary angiography

No deep venous thrombosis (DVT) was found. The D-dimer level was elevated at 1,453 D-dimer units (DDU) ng/mL and subsequent CTPA confirmed the presence of bilateral pulmonary emboli in the left and right upper lobes but greater in the left (Figure 5).

Testing for genetic thrombophilic diseases, including cardiolipin IgG, IgM, IgA, protein C activity, protein S level, antithrombin III functional level, and Factor V Leiden mutation, were all negative.

## Discussion

We report a case of PE diagnosed based on a POCUS exam in a patient with a pulmonary coccidioidomycosis infection and psychiatric symptoms with suspected malingering based on the psychiatric evaluation. PE is defined as an obstruction to pulmonary vasculature as a part of VTE. Clinical courses can range from benign to fatal, depending on the type of PE. Based on severity, PE can be classified as submissive and massive. Submissive is defined as the presence of RV strain, elevated troponin, and brain natriuretic peptide, whereas massive is defined as the presence of thrombus in transit, syncope, cardiac arrest, hemodynamic compromise (systolic BP < 90 mmHg, drop > 40 mmHg, or use of vasopressors).

In our case, the nonspecific signs and symptoms of PE along with the potential malingering could have delayed the diagnosis and led to sentinel events. However, POCUS quickly raised suspicion about an unexpected diagnosis. A recent meta-analysis by Fields et al. showed that RV strain in PE has a sensitivity of 53% and specificity of 83%, making it an accurate, quick, and safe rule-in bedside test [9]. The absence of RV strain on cardiac POCUS does not help with ruling PE out. Similar results were found in a 2021 meta-analysis in which the D-sign had a sensitivity of about 30% but a specificity of 96% [10]. Another systematic review found that lung ultrasound has a sensitivity of 87% and specificity of 81.8% for PE [11]. Subpleural pulmonary consolidation (typically triangular [like our case] or rounded) due to PE was found in more than 75% of PE cases. One prospective study in the ED found that the triple POCUS exam (cardiac, lung, and leg veins ultrasound for DVT) in patients with a Wells score of 5 or more and positive D-dimer has a sensitivity of 90% and specificity of 86.2% for PE, and it also helped in finding an alternative diagnosis in one-third of patients without PE [12]. Similar results of this combined cardiopulmonary ultrasound exam (without leg veins DVT exam) were also obtained with a pooled sensitivity of 91% and specificity of 81% compared to CTPA. The Society of Hospital Medicine and the American College of Physicians both acknowledge the expanding use of POCUS by internists for a variety of common applications, including cardiopulmonary manifestations.

Coccidioidomycosis, on the other hand, is a fungal infection caused by C. posadasii and C. immitis. Infection can range from asymptomatic to disseminated infection involving the skin, pulmonary, or meningeal involvement. The incidence of coccidioidomycosis has increased in the past blamed to the increasing population in central California as well as Arizona. An increase in immunocompromised individuals and constructions in the desert zones also contributed to the increased incidence [13]. African American patients (similar to our case) are known to have an increased risk for infection as well as those with Filipino ancestry.

A case series of five patients diagnosed with coccidioidomycosis in association with VTE complicated with PE was published in 2020 [8]. All the cases were from the Central Valley encountered in a single year. According to the authors, such an association was never reported in the past, the underlying cause of which is not well known. Possible explanations for this include decreased immobility with the onset of the disease coupled with a heightened immune response that may cause endothelial injury and hypercoagulability in the blood, which increases the risk of thrombus formation. Nevertheless, one of the five patients developed DVT, which cannot be explained by pulmonary pathology alone. The five patients also were not significantly immobilized before their presentations. Our patient also did not have any known acquired or genetic susceptibility and was young and otherwise healthy and mobile before presentation, which makes it unique as well. In addition, a case of massive dural and cerebral venous thrombosis associated with coccidioidomycosis meningitis was reported in a patient with AIDS [14]. Taken together, these cases underscore the importance of quickly detecting VTE, given the unusual propensity for developing VTE with different types of coccidioidomycosis infection.

## Conclusions

When encountering patients with chest pain and respiratory complaints, PE is often on the list of differential diagnoses and CTPA is the gold standard test to make this diagnosis. Obtaining and interpreting a chest CTPA involves exposing our patients to harmful radiation and contrast, and there is often a delay in obtaining results, especially in resource-poor settings. We demonstrated here that cardiac and lung POCUS had significant utility and ease of use in raising the clinical suspicion toward PE, especially when clinical suspicion is not high and another confounding diagnosis (like malingering in our case) is being entertained. Moreover, as there are only very few reported cases of association between PE and pulmonary coccidioidomycosis, the appropriate diagnosis in the case was low. Similar to the only reported case series we found, this case also reports an association between these two entities that need further research. By reporting this case, we hope to expand the pathologic spectrum of pulmonary coccidioidomycosis infection and gain further insights into the pathophysiological perspective behind the association of infectious disease and VTE.
